# Associations of androgens with depressive symptoms and cognitive status in the general population

**DOI:** 10.1371/journal.pone.0177272

**Published:** 2017-05-12

**Authors:** Hanna Kische, Stefan Gross, Henri Wallaschofski, Hans Jörgen Grabe, Henry Völzke, Matthias Nauck, Robin Haring

**Affiliations:** 1 Institute of Clinical Chemistry and Laboratory Medicine, University Medicine Greifswald, Greifswald, Germany; 2 German Centre for Cardiovascular Research (DZHK), partner site Greifswald, Greifswald, Germany; 3 Department of Cardiology, University Medicine Greifswald, Greifswald, Germany; 4 Department of Psychiatry and Psychotherapy, University Medicine Greifswald, Greifswald, Germany; 5 Institute for Community Medicine, University Medicine Greifswald, Greifswald, Germany; 6 European University of Applied Sciences, Faculty of Applied Public Health, Rostock, Germany; 7 School of Public Health and Preventive Medicine, Monash University, Melbourne, Australia; Baylor College of Medicine, UNITED STATES

## Abstract

**Objectives:**

Associations between androgens and depressive symptoms were mostly reported from cross-sectional and patient-based studies.

**Study design/main outcome measures:**

Longitudinal data from 4,110 participants of the Study of Health in Pomerania were used to assess **se**x-specific associations of baseline total and free testosterone, androstenedione and sex hormone-binding globulin with incident depressive symptoms and cognitive status at 5- and 10-year follow-up.

**Results:**

Despite sex-specific differences in depressive symptoms prevalence at baseline (women: 17.4%, men: 8.1%), cross-sectional analyses showed no associations between sex hormones and depressive symptoms. In age-adjusted longitudinal analyses, total testosterone was associated with incident depressive symptoms (relative risk at 5-year follow-up: 0.73, 95% confidence interval: 0.58–0.92). Similarly, age-adjusted analyses showed a positive association between sex hormone-binding globulin and cognitive status in men (β-coefficient per standard deviation: 0.44, 95% confidence interval: 0.13–0.74). In women, age-adjusted associations of androstenedione with baseline depressive symptoms (relative risk: 0.88, 95% confidence interval: 0.77–0.99) were found. None of the observed associations remained after multivariable adjustment.

**Conclusions:**

The present population-based, longitudinal study revealed inverse associations between sex hormones and depressive symptoms. However, the null finding after multivariable adjustment suggests, that the observed associations were not independent of relevant confounders including body mass index, smoking and physical inactivity. Furthermore, the low number of incident endpoints in our non-clinical population-based sample limited the statistical power and reduced the chance to detect a statistically significant effect.

## Introduction

Depression is widely recognized as a major health problem and potential risk factor for increased morbidity and decreased quality of life. The observed sex difference in depressive symptoms prevalence motivated research efforts to investigate the role of sex hormones in this gender gap [1]. Since the brain is both, a regulator for sex hormone production and a target for sex hormones crossing the blood-brain barrier, this interplay regulates physiologic processes and may contribute to modulate certain aspects of cognition, mood and socio-emotional functioning [2].

However, to date, epidemiological and clinical studies on depressive symptoms in women have yielded inconsistent results, showing both, positive and inverse associations between androgens and depression or depressive symptoms.

In men, associations between testosterone and depressive symptoms were observed in specific subpopulations and cross-sectional samples [3], lacking generalizability and conclusions about cause and effect. Consequently, both directions of associations were reported, with depressed males exhibiting lower testosterone concentrations compared to healthy controls [4], as well as free testosterone (fT) being related to adverse Beck Depression Inventory scores in older men [5]. Reported associations between low fT [3,6] and low total testosterone (TT) [7,8] and prevalence and incidence of depression or depressive symptoms in men were mainly reported in the elderly. Contrarily, even in older men a study on androgen concentrations and androgen receptor polymorphism did not support a link between androgens and depressive symptoms based on the Geriatic Depression Scale [9].

Although it is well known, that cognitive functions [2] and androgen concentrations decrease with age [10], definite associations between androgens and cognitive status are far from well understood. Previous observational research suggests an association between fT concentrations and cognitive decline [2,11] and a neuroprotective effect of testosterone in older men [12]. Also women under testosterone treatment showed stable cognitive test scores, compared to untreated women in the control group [13]. In a randomized, double-blind, placebo-controlled clinical trial it is suggested that hormone therapy influences changes in specific cognitive functions [14].

However, given the preliminary evidence from previous observational studies, limited by an older study population, immunoassay-based measurements or cross-sectional study design, the aim of our study was to investigate cross-sectional and longitudinal associations of a large androgen panel and sex hormone-binding globulin (SHBG) with depressive symptoms and cognitive status in men and women from the general population.

## Methods

### Study population

The Study of Health in Pomerania (SHIP) is a population-based cohort study in north-eastern Germany. We previously published details of the study design, recruitment, and procedures [15]. In brief, from the target population of individuals with German citizenship and main residency in the study area of West Pomerania comprising 213,057 inhabitants in 1996, a two-stage stratified cluster sample of adults aged 20–79 years was drawn. The net sample comprised 6,265 eligible individuals (3,160 women). Of these, 4,308 individuals (2,192 women) lastly participated between 1997 and 2001 in the baseline study (response 68.8%), after written informed consent was obtained from each participant. Five-year follow-up examinations were conducted between 2002 and 2006 (SHIP-1), involving 3,300 individuals (1,711 women, response: 83.6%) and 10-year follow-up examinations between 2008 and 2012 (SHIP-2) involving 1718 participants (1,235 women, response 54.2%). Ethics Committee of the University of Greifswald authorized the study protocol, which is consistent with the principles of the Declaration of Helsinki. We excluded individuals who (overlap exists): received prescribed drugs in the last seven days, based on the medication packages or on self-statement, categorized based on the Anatomical Therapeutic Chemical classification index (sex hormone antagonists (N = 2 women, 135 men) and natural opium alkaloids (N = 33 women, 19 men)) as well as pregnant women (N = 9). Finally, we investigated a study population of 4,110 participants (2,148 women). Analyses of cognitive status were performed among 366 participants (184 women) with complete data on mini-mental state examination (MMSE).

### Measures

We assessed sociodemographic and behavioral characteristics and medical history conducting a computer-assisted personal interview. Mean daily alcohol consumption was calculated using beverage-specific pure ethanol volume proportions. Participants were considered being physically inactive, when they participated in physical training less than one hour a week during winter or summer. Participants were divided into three categories of smoking habits: current, former, and never-smokers. The sample was stratified into pre- and post-menopausal women, applying a previously published categorization: all women <40 years of age and between 40 and 60 years who reported menstrual cycle were classified as pre-menopausal and all women ≥60 years of age, together with all women between 40 and 60 years who reported no menstrual cycle were classified as post-menopausal [16]. Participants were weighted wearing light clothing and no shoes using standard digital scales and height was measured with a digital ultrasound instrument. Waist circumference was measured utilizing a tape midway between the lower rib margin and the iliac crest in the horizontal plane rounded to the nearest mm. Systolic and diastolic blood pressure were measured after a resting period of at least five minutes and three times on the right arm of seated subjects using an oscillometric digital blood pressure monitor (HEM-705CP, Omron Corporation, Tokyo, Japan). Hypertension was defined as blood pressure ≥140/90 mmHg or use of antihypertensive medication. The following socio-demographic variables were assessed by a computer-assisted personal interview and categorized: educational status (<10, 10, or >10 years of schooling), occupational status (Standard International Occupational Prestige Scale), and cohabitation (married or living alone).

### Outcome measures

The primary outcome, depressive symptoms, was defined by two self-reported questionnaire items of the Composite International Diagnostic-Screener (CID-S), based on core diagnostic questions from the Composite International Diagnostic Interview: Have you experienced more than one period in your life in which 1) you felt more than two weeks almost daily sad or depressed? 2) you suffer more than two weeks almost daily from loss of interest, fatigue or shiftlessness? If question 1) and 2) were answered positively, the participant was classified as depressive [17]. The secondary outcome, cognitive status, based on the questionnaire-based 30-item, MMSE with scores ≤9 indicating severe, moderate (10–18) or mild (19–23) cognitive impairment, and normal cognition (≥24), respectively.

### Laboratory measurements

A detailed description of the performed TT and ASD measurements was previously published [18]. Briefly, serum aliquots (storage at -80°C) consisted of a blood sample, which was taken from the cubital vein in the supine position between 8:00 a.m. and 7:00 p.m. Measurements of serum TT and ASD concentrations were carried out between December 2010 and March 2011 in the Department of Clinical Chemistry at the University Hospital of South Manchester (Manchester, UK). Liquid chromatography-tandem mass spectrometry (LC-MS/MS) with a validated routine method was performed [19], with the result that the standard curve was linear to 50.0 nmol/L, the lower limit of quantitation was 0.25 nmol/L, and intra- and inter-assay coefficients of variation were <10% for both TT and ASD over the range 0.3–35 nmol/L. SHBG concentrations were measured from frozen serum aliquots using a radioimmunoassay on an Advia Centaur (Siemens, Eschborn, Germany) with an inter-assay coefficient of variation of 6.6% at the 27.1 nmol/L level, 7.6% at the 48.2 nmol/L level, and 7.7% at the 52.3 nmol/L level. FT was calculated: [free T (nmol/L) = ((-a+$\sqrt{b}$)/c)/10^−9^ with a = SHBG (nmol/L) -TT (nmol/L) +23.43, b = a^2^ + (4*23.43*TT (nmol/L))] and c = 2*23.43*10^9^ for a standard average albumin concentration of 4.3 g/dL [20]. Androgens were only measured at baseline.

### Statistical analysis

Categorical data are given as percentage and continuous data as mean (standard error) or median (p25^th^, p75^th^). Continuous hormone concentrations were naturally log transformed to normalize their distributions and categorized by age-specific quartiles (for each 10-year age group). First, we implemented age- and multivariable-adjusted generalized Poisson regression models with robust standard errors to examine cross-sectional and longitudinal associations of TT, fT, ASD, and SHBG with depressive symptoms. Effects were presented as relative risk (RR) together with 95% confidence interval (CI) for continuous (increase per standard deviation) and categorized (with quartile one as reference) analyses. The P-value for trend was calculated by involving androgen quartiles as an ordinal score into the regression model. In longitudinal analyses, the sample was restricted to individuals without prevalent depressive symptoms at baseline. Next, we analyzed associations of androgens and SHBG concentrations with cognitive status (change in mini mental status) using linear regression models. Effects are presented as β-coefficients together with 95% CI. We used QQ-plots to test the normality of regression residuals. The multivariable model was adjusted for age, body mass index, smoking status (three categories), alcohol consumption, physical inactivity and hypertension. Additionally, an extended multivariable model included adjustment for occupational status, educational level, and civil status. When analyzing the change in mini mental status, we adjusted all models additionally for baseline MMSE. Multiplicative interaction terms between each hormone and covariate was investigated in multivariable models. Given a number of significant interactions terms (p-value <0.05), all analyses were performed sex-specific. During sensitivity analyses, multivariable regression models among women were stratified for hormone therapy (women using hormone therapy N = 445), OC use (women using OC, N = 430) and menopausal status (pre-menopausal women N = 1132, post-menopausal women N = 1013) and time and date of blood sampling in all individuals. Furthermore, we excluded all participants using antidepressants (women N = 39, men N = 17), women with self-reported hysterectomy/bilateral oophorectomy (N = 230 women) and additionally adjusted all analyses of androgens for SHBG. Due to sample attrition over the 10-year follow-up period, we also adjusted regression models for possible drop-out bias, by using inverse probability weights. To replicate previously reported findings from selected study populations [11,21], multivariable analyses were repeated in specific subgroups according to age and comorbidity. P-values <0.05 were considered statistically significant. All statistical analyses were performed with Stata 13.0 (Stata Corp., College Station, TX, USA).

## Results

**Fig 1:**
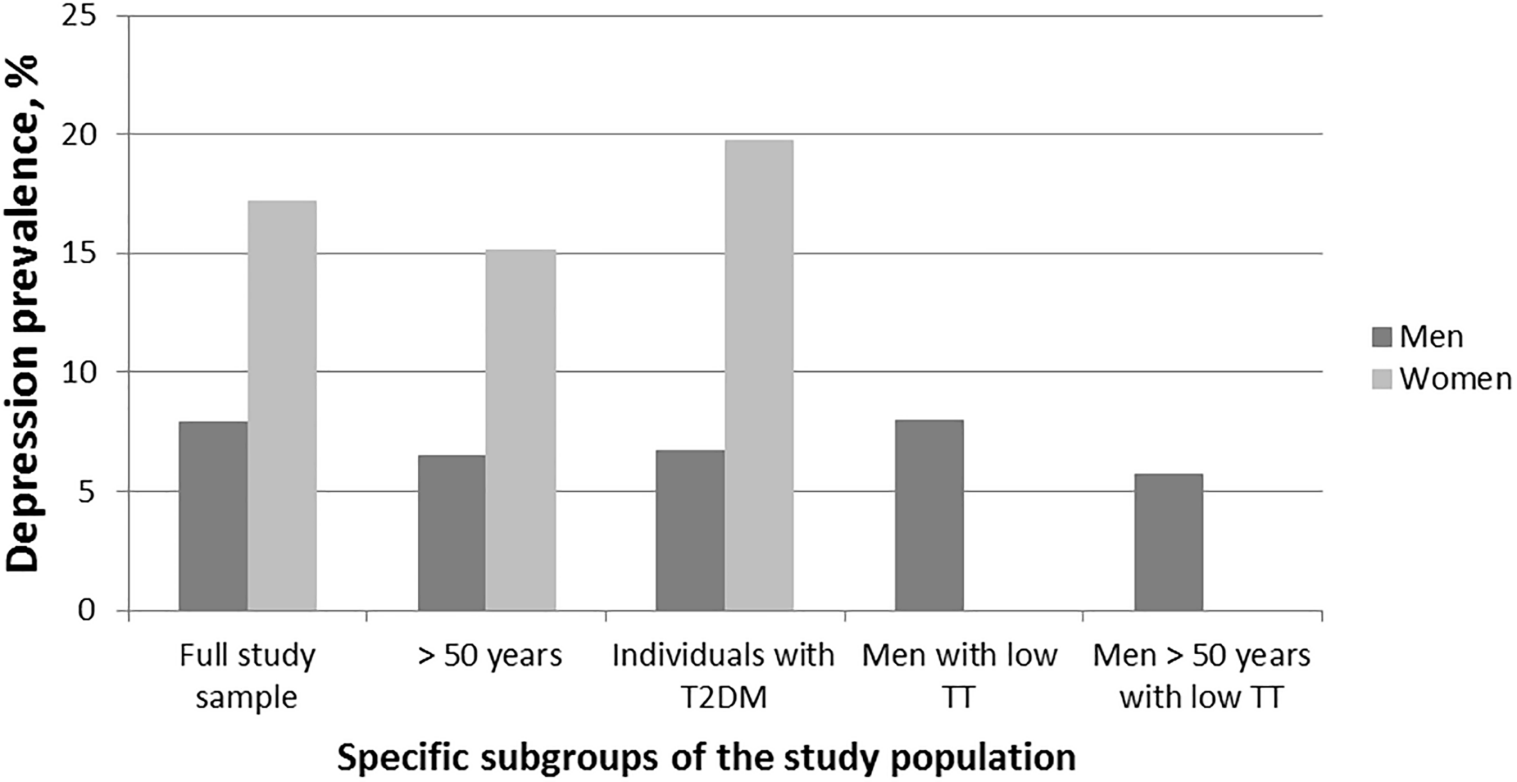
Depressive symptoms prevalence at baseline by specific subgroups. Low total testosterone was defined as <10.4 nmol/L. TT, total testosterone; T2DM, type 2 diabetes mellitus.

Table 1 presents the baseline characteristics of the full study sample, stratified by sex. With respect to cardiometabolic variables, women showed lower BMI and waist circumference, as well as lower systolic and diastolic blood pressure, compared to men (p < 0.01). The primary outcome depressive symptoms showed a sex-specific prevalence (women: 17.4%, men: 8.1%). The incidence of depressive symptoms in 5- and 10-year follow-up was 6,5% and 6,7% in women and 3,7% and 2,9% in men, respectively. Among older men (>50 years) with low TT concentrations (<10.4 nmol/L), the depressive symptoms prevalence of 5.8% was lower compared to the full male study population (Fig 1).

**Table 1:** Baseline characteristics of the study population, stratified by sex.

Variable	Women (N = 2148)	Men (N = 1962)	P*
**Age, years**	48.8 (16.1)	49.5 (16.3)	0.14
**Age range, years**	20–81	20–81	-
**Total Testosterone, nmol/L**	0.8 (0.6; 1.1)	14.8 (11.4; 19.1)	< 0.01
**SHBG, nmol/L**	83.2 (57.9; 123.9)	46.5 (34.1; 61.2)	< 0.01
**Calculated Free Testosterone, nmol/L**	0.006 (0.004; 0.01)	0.24 (0.19; 0.30)	< 0.01
**Androstenedione, nmol/L**	1.6 (1.0; 2.5)	1.9 (1.4; 2.5)	< 0.01
**Body Mass Index, kg/m**^**2**^	26.9 (5.3)	27.6 (4.0)	< 0.01
**Waist circumference, cm**	83.1 (13.1)	95.5 (11.7)	< 0.01
**Current smoking, %**	26.9	35.6	< 0.01
**Physically inactive, %**	56.5	58.3	0.2
**Alcohol consumption, g/day**	3.1 (0.7; 7.8)	11.4 (3.3; 26.8)	< 0.01
**Oral contraceptive use, %**	20.2	**-**	**-**
**Hormone replacement therapy, %**	20.7	**-**	**-**
**Systolic blood pressure, mmHg**	129.9 (21.3)	142.4 (19.4)	< 0.01
**Diastolic blood pressure, mmHg**	80.9 (10.7)	86.1 (11.4)	< 0.01
**Antihypertensive medication, %**	24.3	24.9	0.6
**Hypertension, %**	42.1	62.3	< 0.01
**Antidepressive medication, %**	2.7	1.2	0.01
**Depressive symptoms, N**			
**Baseline**	370 (17.2)	156 (7.9)	< 0.01
**5-year follow-up**	166 (10.0)	73 (5.0)	< 0.01
**10-year follow-up**	114 (9.5)	63 (3.5)	< 0.01
**Mini mental status, N, score**			
**Baseline**	588; 26 (24; 28)	572; 26 (23; 28)	0.5
**5-year follow-up**	634; 28 (26;29)	573; 28 (27;29)	0.3
**10-year follow-up**	521; 29 (27;30)	465; 29 (27;29)	0.4

Data are absolute numbers (percentages), mean (SD) or median (Q1, Q3). Oral contraceptive use was defined according to Anatomical Therapeutic Chemical (ATC) classification code G03AA/B/C/D. Values of sex hormones are reported based on availability of each hormone. Total testosterone: women N = 1642, men N = 1555; SHBG: women N = 1982, men N = 1826; androstenedione: women N = 1704, men N = 1517; free testosterone: women N = 1525, men N = 1448.

*Statistical comparisons were performed with χ 2 test (nominal data) or Mann-Whitney-U-test (continuous data).

SHBG, sex hormone-binding globulin.

In men, cross-sectional analyses revealed no significant associations between androgens and depressive symptoms in men (Table 2). Longitudinal analyses showed inverse associations between continuous TT concentrations and depressive symptoms during 5-year (RR per SD: 0.73, 95% CI: 0.58–0.92), as well as 10-year follow-up (RR: 0.71, 95% CI: 0.56–0.92) in age-adjusted, but not in multivariable models. Similarly, higher TT was inversely associated with incident 5-year depressive symptoms (Q1 (Ref.) vs. Q4, RR: 0.26, 95% CI: 0.07; 0.91). In women, cross-sectional and longitudinal analyses revealed no associations between androgens and depressive symptoms after multivariable adjustment (Table 2). Similarly, categorized analyses showed no significant trend across androgen quartiles.

**Table 2:** Associations of sex hormones and SHBG with depressive symptoms in Poisson regression models, separately in men and women.

	Relative Risk (95% CI)							
	Total Testosterone	Androstenedione	Free Testosterone	SHBG	Total Testosterone	Androstenedione	Free Testosterone	SHBG
**Men**					**Women**			
**cross-sectional, age-adjusted**								
continuous	1.05 (0.85; 1.23)	1.02 (0.85; 1.22)	1.00 (0.84; 1.20)	1.02 (0.86; 1.22)	0.89 (0.79; 1.00)	0.88 (0.77; 0.99)*	0.94 (0.84; 1.05)	1.02 (0.93; 1.12)
1. Quartile	Ref.	Ref.	Ref.	Ref.	Ref.	Ref.	Ref.	Ref.
2. Quartile	0.80 (0.49; 1.29)	0.93 (0.55; 1.57)	1.05 (0.65; 1.68)	0.79 (0.50; 1.23)	1.01 (0.77; 1.34)	1.03 (0.78; 1.36)	1.22 (0.90; 1.64)	0.99 (0.74; 1.32)
3. Quartile	0.80 (0.49; 1.30)	1.16 (0.70; 1.90)	0.69 (0.40; 1.18)	0.76 (0.48; 1.19)	0.80 (0.60; 1.08)	0.80 (0.60; 1.08)	0.90 (0.65; 1.25)	1.34 (1.03; 1.75)
4. Quartile	0.98 (0.62; 1.55)	1.09 (0.66; 1.81)	0.95 (0.58; 1.55)	0.99 (0.66; 1.51)	0.74 (0.54; 1.01)	0.90 (0.67; 1.20)	0.93 (0.67; 1.29)	1.04 (0.78; 1.38)
**cross-sectional, multivariable-adjusted**								
continuous	0.96 (0.82; 1.13)	1.09 (0.89; 1.33)	1.00 (0.84; 1.19)	0.96 (0.80; 1.15)	0.90 (0.80; 1.02)	0.88 (0.77; 1.00)	0.95 (0.84; 1.08)	1.00 (0.90; 1.11)
1. Quartile	Ref.	Ref.	Ref.	Ref.	Ref.	Ref.	Ref.	Ref.
2. Quartile	0.78 (0.47; 1.27)	0.93 (0.54; 1.62)	1.03 (0.63; 1.67)	0.78 (0.50; 1.22)	0.98 (0.74; 1.31)	1.05 (0.79; 1.39)	1.19 (0.88; 1.62)	0.91 (0.67; 1.24)
3. Quartile	0.66 (0.39; 1.12)	1.22 (0.73; 2.03)	0.61 (0.35; 1.08)	0.71 (0.45; 1.12)	0.79 (0.74; 1.08)	0.82 (0.61; 1.12)	0.89 (0.63; 1.25)	1.25 (0.94; 1.66)
4. Quartile	0.82 (0.49; 1.38)	1.24 (0.72; 2.14)	0.95 (0.56; 1.58)	0.85 (0.54; 1.33)	0.75 (0.55; 1.03)	0.91 (0.67; 1.23)	0.96 (0.68; 1.36)	0.98 (0.72; 1.33)
**5-year follow-up, age-adjusted**								
continuous	0.73 (0.58; 0.92)*	0.62 (0.42; 0.92)*	0.75 (0.55; 1.01)	0.85 (0.62; 1.18)	0.90 (0.70; 1.15)	0.93 (0.67; 1.27)	0.93 (0.72; 1.22)	1.11 (0.90; 1.36)
1. Quartile	Ref.	Ref.	Ref.	Ref.	Ref.	Ref.	Ref.	Ref.
2. Quartile	0.71 (0.30; 1.69)	0.77 (0.34; 1.70)	0.84 (0.34; 2.04)	0.54 (0.25; 1.25)	0.58 (0.27; 1.22)	0.85 (0.45; 1.62)	1.15 (0.59; 2.24)	1.06 (0.54; 2.06)
3. Quartile	0.96 (0.43; 2.11)	0.39 (0.14; 1.08)	0.84 (0.34; 2.04)	0.58 (0.26; 1.29)	0.91 (0.49; 1.70)	0.64 (0.31; 1.29)	0.60 (0.27; 1.32)	2.04 (1.13; 3.68)*
4. Quartile	0.26 (0.07; 0.91)*	0.34 (0.11; 1.04)	0.58 (0.21;1.57)	0.77 (0.36; 1.63)	0.73 (0.37; 1.43)	0.92 (0.49; 1.74)	0.96 (0.48; 1.93)	1.37 (0.74; 2.55)
**5-year follow-up, multivariable-adjusted**								
continuous	0.75 (0.57; 1.00)	0.67 (0.45; 1.00)	0.77 (0.54; 1.09)	0.89 (0.61; 1.30)	0.91 (0.70; 1.18)	0.97 (0.70; 1.34)	0.95 (0.72; 1.26)	1.08 (0.89; 1.32)
1. Quartile	Ref.	Ref.	Ref.	Ref.	Ref.	Ref.	Ref.	Ref.
2. Quartile	0.67 (0.27; 1.64)	0.85 (0.38; 1.93)	0.84 (0.33; 2.10)	0.51 (0.22 (1.18)	0.53 (0.24; 1.14)	0.89 (0.45; 1.73)	1.04 (0.52; 2.09)	0.93 (0.48; 1.08)
3. Quartile	1.13 (0.49; 2.62)	0.45 (0.15; 1.31)	1.02 (0.42; 2.45)	0.54 (0.22; 1.30)	0.97 (0.51; 1.85)	0.74 (0.36; 1.53)	0.61 (0.28; 1.33)	2.07 (1.17; 3.68)*
4. Quartile	0.32 (0.08; 1.22)	0.41 (0.13; 1.28)	0.73 (0.27; 1.96)	0.82 (0.35; 1.94)	0.73 (0.37; 1.43)	1.03 (0.54; 1.97)	0.95 (0.46; 1.96)	1.24 (0.66; 2.34)
**10-year follow-up, age-adjusted**								
continuous	0.71 (0.56; 0.92)*	0.86 (0.49; 1.50)	0.84 (0.55; 1.27)	0.60 (0.38; 0.95)*	1.05 (0.81; 1.35)	0.87 (0.63; 1.18)	0.88 (0.68; 1.14)	1.18 (0.96; 1.44)
1. Quartile	Ref.	Ref.	Ref.	Ref.	Ref.	Ref.	Ref.	Ref.
2. Quartile	0.48 (0.12; 1.90)	0.93 (0.22; 3.82)	0.89 (0.22; 3.53)	0.41 (0.12; 1.32)	0.90 (0.40; 2.00)	0.84 (0.41; 1.73)	1.15 (0.55; 2.41)	1.30 (0.65; 2.60)
3. Quartile	0.64 (0.18; 2.20)	1.49 (0.40; 5.59)	0.92 (0.24; 3.54)	0.58 (0.19; 1.68)	1.30 (0.65; 2.62)	0.92 (0.45; 1.86)	1.17 (0.56; 2.40)	1.72 (0.88; 3.34)
4. Quartile	0.18 (0.02; 1.56)	0.27 (0.02; 2.73)	0.45 (0.08; 2.44)	0.37 (0.10; 1.38)	1.12 (0.53; 2.35)	0.84 (0.40; 1.75)	0.94 (0.43; 2.05)	1.31 (0.65; 2.61)
**10-year follow-up, multivariable-adjusted**								
continuous	0.72 (0.51; 1.01)	0.97 (0.59; 1.61)	0.85 (0.47; 1.52)	0.64 (0.37; 1.10)	1.04 (0.80; 1.35)	0.86 (0.61; 1.20)	0.87 (0.66; 1.16)	1.22 (0.99; 1.50)
1. Quartile	Ref.	Ref.	Ref.	Ref.	Ref.	Ref.	Ref.	Ref.
2. Quartile	0.58 (0.14; 2.41)	0.90 (0.21; 3.77)	0.74 (0.17; 3.09)	0.51 (0.15; 1.69)	0.90 (0.40; 2.04)	0.78 (0.37; 1.65)	1.13 (0.54; 2.35)	1.47 (0.70; 3.10)
3. Quartile	0.72 (0.16; 3.14)	1.74 (0.47; 6.42)	1.01 (0.24; 4.23)	0.53 (0.17; 1.68)	1.18 (0.58; 2.41)	0.84 (0.40; 1.75)	1.15 (0.55; 2.43)	2.14 (1.05; 4.34)
4. Quartile	0.29 (0.03; 2.70)	0.36 (0.04; 3.20)	0.53 (0.09; 2.99)	0.54 (0.13; 2.31)	1.16 (0.54; 2.49)	0.86 (0.41; 1.80)	0.96 (0.41; 2.22)	1.45 (0.69; 3.02)

Data are relative risk and their 95% confidence interval with p < 0.05 marked as *. Quartile 1 was used as reference.

The multivariable model was adjusted for age, body mass index, smoking status (three categories), alcohol consumption, physically inactivity, and hypertension. Longitudinal analyses were additionally adjusted for inverse probability weights for drop-out of baseline examination to follow-up and performed only in participants without depressive symptoms at baseline.

SHBG, sex hormone-binding globulin; CI, confidence interval.

**Fig 2:**
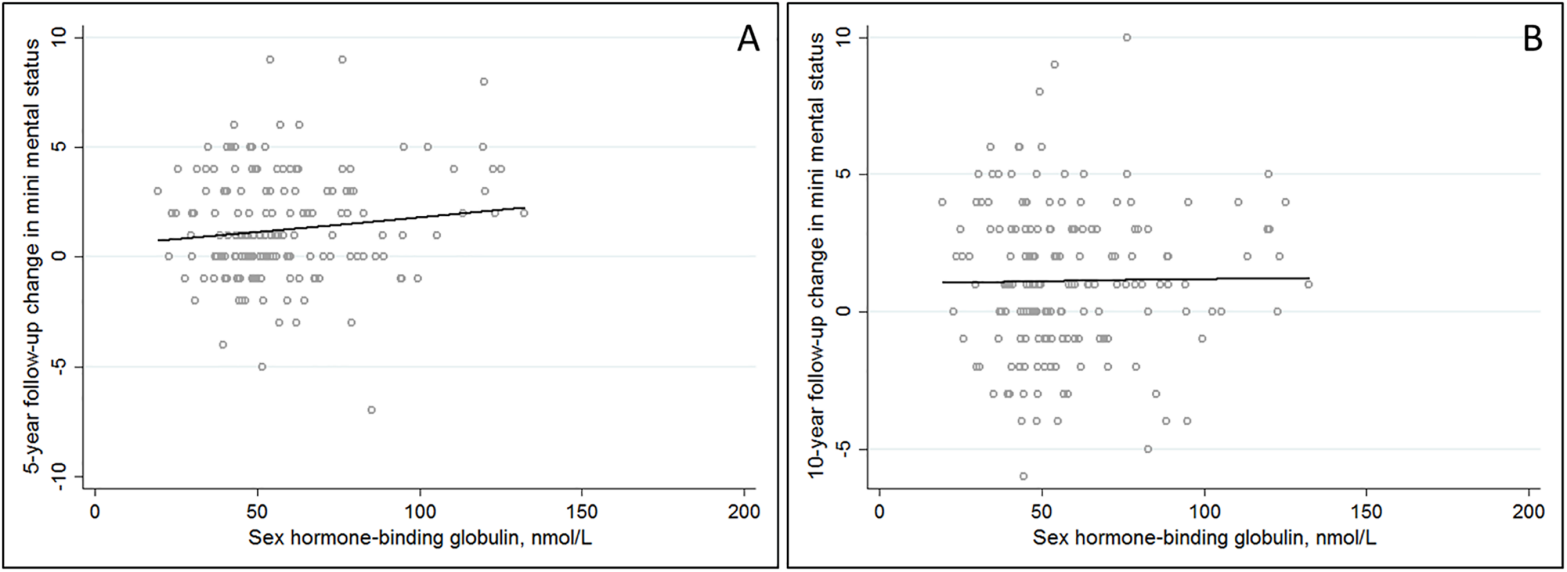
Scatterplot for sex hormone-binding globulin and change in mini mental status in men at 5-year follow-up (A) and 10-year follow-up (B). Age-adjusted analyses revealed a significant, positive association between sex hormone-binding globulin and 5-year change in mini mental status (β-coefficient: 0.44, 95% CI: 0.13–0.74, p = 0.03).

With regard to the secondary outcome, we observed a positive association between SHBG and 5-year change in MMSE among men in age-adjusted models (β-coefficient: 0.44, 95% CI: 0.13–0.74) (Fig 2).

After multivariable-adjustment we found no associations between androgens and change in MMSE in both sexes (Table 3). Stratified analyses by menopausal status, oral contraceptiva use, hormone therapy, and antidepressants did not reveal any statistical evidence for relevant subgroup differences. Multivariable analyses among specific disease subgroups also yielded no substantial change in the overall estimates (S1 Table). Finally, additional adjustment for time and date of blood sampling did not substantially alter the revealed estimates (S2 Table and S3 Table).

**Table 3:** Associations of sex hormones and SHBG with change in mini mental status in linear regression models, separately in men and women.

	β-coefficients (95% CI)			
	Total Testosterone	Androstenedione	Free Testosterone	SHBG
**Men**				
**5-year follow-up**				
age-adjusted	0.13 (-0.11; 0.38)	0.35 (-0.20; 0.91)	0.03 (-0.19; 0.26)	0.44 (0.13; 0.74)*
multivariable-adjusted	0.02 (-0.15; 0.20)	0.38 (-0.16; 0.93)	-0.01 (-0.21; 0.19)	0.23 (-0.11; 0.59)
**10-year follow-up**				
age-adjusted	0.01 (-0.20; 0.23)	0.32 (-0.14; 0.80)	0.07 (-0.16; 0.31)	0.10 (-0.26; 0.47)
multivariable-adjusted	0.01 (-0.22; 0.24)	0.27 (-0.21; 0.75)	0.05 (-0.20; 0.30)	0.08 (-0.30; 0.46)
**Women**				
**5-year follow-up**				
age-adjusted	-0.03 (-0.44; 0.36)	-0.17 (-0.48; 0.13)	0.23 (-0.19; 0.66)	-0.31 (-0.74; 0.10)
multivariable-adjusted	-0.10 (-0.49; 0.27)	-0.23 (-0.56; 0.09)	0.23 (-0.17; 0.64)	-0.37 (-0.80; 0.04)
**10-year follow-up**				
age-adjusted	0.06 (-0.37; 0.50)	0.01 (-0.31; 0.35)	0.24 (-0.30; 0.80)	-0.10 (-0.48; 0.26)
multivariable-adjusted	-0.03 (-0.47; 0.41)	0.01 (-0.37; 0.41)	0.14 (-0.45; 0.75)	-0.08 (-0.46; 0.28)

Data are β coefficients and their 95% confidence interval with p < 0.05 marked as *.

The multivariable model was adjusted for age, body mass index, smoking status (three categories), alcohol consumption, physically inactivity, hypertension, baseline mini mental status, and inverse probability weights for drop-out of baseline examination to follow-up. 5-year follow-up change in mini mental status: women N = 176, men N = 177. 10-year follow-up: women N = 184, men N = 182.

SHBG, sex hormone-binding globulin; CI, confidence interval.

## Discussion

The present population-based study revealed no statistically significant associations between androgens and depressive symptoms in cross-sectional and longitudinal analyses. Additionally the non-significant findings after multivariable-adjustment highlight the relevant role of cofactors in the association between androgens and depressive symptoms.

The present null finding is in line with previous observational cohorts like the Rancho Bernardo study [22] and relevant meta-analysis of population-based studies [23]. In contrast to previous findings from selected samples, we observed no statistically significant associations during sensitivity analyses in subgroups of elderly men with low TT or various comorbid conditions. Interestingly, even the depressive symptoms prevalence in these subgroups did not differ compared to the entire study sample.

Longitudinal analyses in men revealed inverse associations between TT and depressive symptoms in 5- and 10-year follow-up, but only in age-adjusted models. The link between low TT and depressive symptoms is described in previous meta-analysis [24] and observational research, which suggest that low TT concentrations are associated with an earlier onset and greater incidence of depressive illness in older men. In contrast, another review observed no significant effect of testosterone replacement on mood in eugonadal males and contradictory results in hypogonadal males [25]. Additionally, several studies observed that older men with depression or men using antidepressant had lower TT concentrations compared to controls [3]. Interestingly, this observation cannot be confirmed in our population-based sample. These differences may be due to selection of participants and especially the study-dependent age-range. Further, it is possible that associations between low TT and depressive symptoms may depend upon androgen receptor genetic polymorphisms, thus variability in receptor transactivation may contribute to variability in the expression of androgen-mediated psychiatric symptoms as has been suggested by results of The Massachusetts Male Aging Study (MMAS) [26]. At this, in men with shorter CAG repeat length—a genetic trait marker associated with androgen receptor function—low versus high testosterone was associated with a five-fold increased likelihood of depressive symptoms. In regard of cofactors, all associations between androgens and depressive symptoms in our study are only significant in age-adjusted but not in multivariable models, confirming the impact of lifestyle-related and social factors as well as comorbidities on the associations between androgens and depressive symptoms [27]. Overall, these findings should be interpreted in light of the characteristics of the study sample. Given the low incidence of depressive symptoms, the statistically non-significant associations in longitudinal, multivariable-adjusted analyses could also be due to a potential lack of statistical power.

In women, we observed a borderline significance for the inverse association between ASD and baseline depressive symptoms. Not only in men, but also in women androgen concentrations decrease continuously with age with about to 50% before menopause compared to 20-year-old women. A population-based study among German women showed that symptoms as depressive mood increased from 20% in 18–29 years old, to 48% for 50–59 year old [28]. Associations between specific androgens and depressive symptoms were mainly investigated in regard of testosterone. A link between the increase in TT concentrations in pubertal girls and in women suffering from postpartum “baby blues” [29] and depressive symptoms was found. In this study, the hypothesis that testosterone in women is correlated with depressive symptoms suggested by some previous research was not confirmed. Nevertheless, our results regarding testosterone in women are in line with other large population-based studies i.e. the Rancho Bernardo study [30]. Since findings among association between androgens and depressive symptoms are still contradictory, additional double blind, placebo-controlled, randomized clinical trials are warranted.

In terms of the secondary outcome cognitive status, we found no associations between androgens and change in mini mental status in both sexes. The effect of testosterone on cognitive performance is not yet clear. On one side and in line with our findings, long-term testosterone-administration in older men in a recent randomized, double blind, placebo-controlled trial, did not improve cognitive function [31]. On the other hand, testosterone-treatment to non-dement individual was associated with better visuospatial functioning and deteriorations of verbal skills [32]. Additionally, in older women, higher testosterone concentrations predicted better categorical performance on cognitive tests [30]. Overall, the examination of androgens and their potential link to cognitive status yielded widely spread findings, from no associations through to strong significant associations. This might be mainly due to poor comparability of the studies with very different characteristics of the study population and particularly inconsistent definition of cognitive status. Thus, it would be worthwhile to enhance the comparability of prospective studies on cognitive status.

Only in men and in line with previous research [33], we observed an association between SHBG and 5-year change in mini mental status, fading significance in multivariable models. We expected loss of significance when adjusting for waist circumference, since SHBG is correlated with obesity with decreased concentrations in adiposity. Further, there is a growing body of evidence, that cognitive status is associated with obesity as well [33]. Both, SHBG and cognitive status are additionally associated with insulin resistance. Thus, we expected an inverse association between SHBG and cognitive status, but found the opposite. However, also previous studies reported that increased SHBG concentrations represent a risk for cognitive decline and were associated with low verbal memory scores in the elderly [34]. This potential association between SHBG and cognitive status merits further investigation in independent population-based samples.

### Strengths and limitations

Important strengths of our study are the availability of a large population-based sample and the longitudinal study design. An additional strength is the androgens measurement by LC-MS/MS instead of immunoassay, providing a reliable assessment of androgen status, especially in the low concentrations range of women.

This study has several limitations that could affect the generalizability of these findings. We defined depressive symptoms only by CID-S, but this time-efficient diagnostic screening tool of depressive symptoms is commonly used in large population-based samples. In this study, we observed that prevalence of depressive symptoms in longitudinal analyses was low, compared with baseline prevalence of depressive symptoms (5-year follow-up: women 9.9%, men 5.0%; 10-year follow-up: women 9.5%, men 3.5%). This could be explained with the healthy responder effect, assuming that individuals who respond and especially repeatedly respond in follow-up to the examination invitation are healthier than individuals who did not respond. Thus, there is a risk that the study is falsely negative due to high attrition rate among individuals with depressive symptoms. However, previous studies describing prevalence of depression in both old [35] and young [36] population groups revealed a wide variability. Further limitations might arise from the definition of the secondary outcome, since especially onetime measurements in MMSE should be interpreted in light of participant’s age and level of education. Thus, we carefully adjusted all analyses for age and presented a second multivariable model with level of education as cofactor, but without any change in the revealed estimates. Blood samples were taken between 07:00 a.m. and 04:00 p.m.. To consider potential bias from diurnal variation in hormone concentrations, we carefully adjusted for time of blood sampling, but without detecting any significant impact on the previously revealed estimates. In line with the findings of this sensitivity analysis, a previous investigation in SHIP showed only minor differences in TT levels between serum samples drawn before midday and afternoon, therefore, this variation is expected to be minimal [37]. A further limitation might arise from calculated fT. The accuracy on calculated values of fT depends on the validity of the TT measurement–in this study mass-spectrometry based, precise measurements with slight intra- and inter-laboratory variability. Additionally, direct radioimmunoassay for fT did not perform as well as the calculation in previous studies [38]. Finally, the present findings may be limited by androgen measurements based on blood samples taken during any phase of the menstrual cycle. Since we did not assess the phase of the menstrual cycle, we were not able to adjust for this potential source of bias.

## Conclusion

Among men and women from the general population, androgens and SHBG were not independently associated with depressive symptoms. Since all of the observed significant associations were rendered non-significant after multivariable adjustment, these findings underline the relative importance of body composition and health-related lifestyle for the link between androgens and depressive symptoms. Furthermore, the non-clinical population-based nature of our study sample yielded a low incidence of depressive symptoms and thereby limited statistical power and reduced the chance to detect statistically significant estimates during longitudinal analyses. To further elucidate the exact interplay of androgens, depression and relevant confounders, future investigations in large-scale longitudinal studies are needed.

## Supporting information

S1 Table Multivariable-adjusted associations of sex hormones with depression in specific subpopulations of men. (DOCX)

S2 Table Associations of sex hormones and SHBG with depressive symptoms in Poisson regression models, separately in men and women and additionally adjusted for time of blood sampling. (DOCX)

S3 Table Associations of sex hormones and SHBG with change in mini mental status in linear regression models, separately in men and women and additionally adjusted for time of blood sampling. (DOCX)
