# Supplementary material for: Associations of androgens with depressive symptoms and cognitive status in the general population
Source: PLoS One. 2017 May 12;12(5):e0177272. doi: 10.1371/journal.pone.0177272 (PMC5428943; doi:10.1371/journal.pone.0177272)
Supplement: S1 Table — (DOCX) [file pone.0177272.s001.docx]

| **Model** | **Total Testosterone** | **Free Testosterone** | **Androstenedione** | **SHBG** | **N** | **Results compared to full study sample** |
| --- | --- | --- | --- | --- | --- | --- |
| **Age> 50 years** | 1.08 (0.81; 1.45) | 1.11 (0.81; 1.52) | 0.86 (0.63; 1.18) | 1.02 (0.78; 1.33) | 983 | No substantial change in the overall estimates |
| **Age> 50 years**  **TT < 10.4 nmol/L** | 1.03 (0.75; 1.41) | 1.12 (0.84; 1.50) | 0.83 (0.44; 1.57) | 0.66 (0.29; 1.49) | 175 | No substantial change in the overall estimates |
| **Age > 50 years**  **Type 2 Diabetes Mellitus** | 1.34 (0.71; 2.52) | 1.50 (0.75; 2.97) | 1.10 (0.59; 2.06) | 1.48 ( 0.71; 3.06) | 142 | No substantial change in the overall estimates |
| **Age > 50 years**  **BMI > 30**  **Current smoker** | 0.64 (0.23; 1.79) | - | - | 7.90 (0.84; 74.25) | 50 | No substantial change in the overall estimates |
| **Age > 50 years**  **Hypertension**  **BMI > 30** | 0.98 (0.40; 2.39) | 0.57 (0.25; 1.29) | 1.42 (0.89; 2.28) | 1.55 (0.73; 3.27) | 165 | No substantial change in the overall estimates |
| **Age > 50 years**  **Hypertension**  **BMI > 30**  **Current smoker** | 3.08e-9 (6.04e-10; 1.57e-8) | 0.41 (0.15; 1.37) | - | 0.43 (0.09; 1.94) | 27 | Additional associations between TT and baseline depression in men* |
| **TT < Mean TT** | 0.98 (0.56; 1.74) | 1.00 (0.58; 1.74) | 0.99 (0.74; 1.32) | 1.03 (0.79; 1.35) | 940 | No substantial change in the overall estimates |
| **TT < Mean TT**  **Type 2 Diabetes Mellitus** | 5.65 (1.14; 27.91) | 1.60 (0.16; 15.56) | 0.36 (0.12; 0.98) | 1.43 (0.67; 3.05) | 123 | Additional associations between TT and baseline depression in men |

**S1 Table**

**S1 Table 1:** Multivariable-adjusted associations of sex hormones with depression in specific subpopulations of men.

TT, total testosterone; BMI, Body mass index. . *Change in estimates, but lack of power due to small sample size.
