# Supplementary material for: Associations of androgens with depressive symptoms and cognitive status in the general population
Source: PLoS One. 2017 May 12;12(5):e0177272. doi: 10.1371/journal.pone.0177272 (PMC5428943; doi:10.1371/journal.pone.0177272)
Supplement: S2 Table — (DOCX) [file pone.0177272.s002.docx]

**S2 Table**

**S2 Table:** Associations of sex hormones and SHBG with depressive symptoms in Poisson regression models, separately in men and women and additionally adjusted for time of blood sampling.

|  | **Relative Risk (95% CI)** | | | | | | | |
| --- | --- | --- | --- | --- | --- | --- | --- | --- |
|  | **Total Testosterone** | **Androstenedione** | **Free Testosterone** | **SHBG** | **Total Testosterone** | **Androstenedione** | **Free Testosterone** | **SHBG** |
| **Men** | | | | | **Women** | | | |
| **cross-sectional, age-adjusted** | | | | | | | | |
| continuous | 1.06 (0.84; 1.23) | 1.03 (0.84; 1.22) | 1.00 (0.84; 1.20) | 1.03 (0.85; 1.22) | 0.87 (0.76; 1.01) | **0.89 (0.77; 0.99)*** | 0.96 (0.83; 1.01) | 1.05 (0.95; 1.12) |
| 1. Quartile | Ref. | Ref. | Ref. | Ref. | Ref. | Ref. | Ref. | Ref. |
| 2. Quartile | 0.80 (0.49; 1.29) | 0.93 (0.55; 1.57) | 1.05 (0.65; 1.68) | 0.79 (0.50; 1.23) | 1.01 (0.77; 1.34) | 1.01 (0.76; 1.37) | 1.22 (0.90; 1.64) | 0.97 (0.71; 1.36) |
| 3. Quartile | 0.82 (0.49; 1.33) | 1.14 (0.71; 1.90) | 0.69 (0.40; 1.18) | 0.77 (0.45; 1.18) | 0.80 (0.60; 1.08) | 0.80 (0.60; 1.08) | 0.90 (0.65; 1.25) | 1.30 (1.06; 1.78) |
| 4. Quartile | 0.97 (0.62; 1.56) | 1.09 (0.66; 1.81) | 0.95 (0.58; 1.55) | 0.99 (0.66; 1.51) | 0.71 (0.53; 1.02) | 0.90 (0.67; 1.20) | 0.89 (0.65; 1.32) | 1.04 (0.78; 1.38) |
| **cross-sectional, multivariable-adjusted** | | | | | | | | |
| continuous | 0.96 (0.82; 1.13) | 1.09 (0.89; 1.33) | 1.00 (0.84; 1.19) | 0.93 (0.80; 1.15) | 0.90 (0.80; 1.02) | 0.85 (0.78; 1.00) | 0.95 (0.84; 1.08) | 1.00 (0.90; 1.11) |
| 1. Quartile | Ref. | Ref. | Ref. | Ref. | Ref. | Ref. | Ref. | Ref. |
| 2. Quartile | 0.78 (0.47; 1.27) | 0.93 (0.54; 1.62) | 1.04 (0.62; 1.67) | 0.78 (0.50; 1.22) | 0.95 (0.70; 1.31) | 1.05 (0.79; 1.39) | 1.19 (0.88; 1.62) | 0.86 (0.68; 1.25) |
| 3. Quartile | 0.66 (0.39; 1.12) | 1.22 (0.73; 2.03) | 0.61 (0.35; 1.08) | 0.70 (0.44; 1.12) | 0.76 (0.72; 1.08) | 0.80 (0.63; 1.10) | 0.85 (0.65; 1.27) | 1.26 (0.93; 1.69) |
| 4. Quartile | 0.83 (0.50; 1.39) | 1.25 (0.71; 2.14) | 0.93 (0.53; 1.58) | 0.85 (0.54; 1.33) | 0.74 (0.54; 1.03) | 0.91 (0.67; 1.23) | 0.96 (0.68; 1.36) | 0.98 (0.72; 1.33) |
| **5-year follow-up, age-adjusted** | | | | | | | | |
| continuous | **0.73 (0.58; 0.92)*** | **0.62 (0.42; 0.92)*** | 0.74 (0.54; 1.01) | 0.83 (0.60; 1.18) | 0.90 (0.70; 1.15) | 0.91 (0.67; 1.27) | 0.93 (0.72; 1.22) | 1.11 (0.90; 1.36) |
| 1. Quartile | Ref. | Ref. | Ref. | Ref. | Ref. | Ref. | Ref. | Ref. |
| 2. Quartile | 0.72 (0.29; 1.69) | 0.76 (0.33; 1.70) | 0.84 (0.34; 2.04) | 0.53 (0.25; 1.25) | 0.56 (0.27; 1.22) | 0.85 (0.45; 1.62) | 1.15 (0.59; 2.24) | 1.05 (0.51; 2.03) |
| 3. Quartile | 0.97 (0.43; 2.11) | 0.39 (0.14; 1.08) | 0.84 (0.34; 2.04) | 0.58 (0.26; 1.29) | 0.91 (0.49; 1.70) | 0.61 (0.30; 1.29) | 0.60 (0.27; 1.32) | 2**.04 (1.13; 3.68)*** |
| 4. Quartile | **0.26 (0.07; 0.91)*** | 0.34 (0.10; 1.04) | 0.58 (0.21;1.57) | 0.76 (0.34; 1.63) | 0.73 (0.37; 1.43) | 0.92 (0.49; 1.74) | 0.94 (0.48; 1.93) | 1.32 (0.76; 2.59) |
| **5-year follow-up, multivariable-adjusted** | | | | | | | | |
| continuous | 0.76 (0.57; 1.00) | 0.67 (0.45; 1.00) | 0.76 (0.54; 1.09) | 0.89 (0.61; 1.30) | 0.91 (0.70; 1.18) | 0.97 (0.70; 1.34) | 0.95 (0.72; 1.26) | 1.08 (0.89; 1.32) |
| 1. Quartile | Ref. | Ref. | Ref. | Ref. | Ref. | Ref. | Ref. | Ref. |
| 2. Quartile | 0.67 (0.27; 1.64) | 0.85 (0.38; 1.93) | 0.84 (0.33; 2.10) | 0.51 (0.22 (1.18) | 0.51 (0.23; 1.15) | 0.87 (0.43; 1.75) | 1.04 (0.52; 2.09) | 0.93 (0.48; 1.08) |
| 3. Quartile | 1.15 (0.49; 2.64) | 0.44 (0.16; 1.32) | 1.03 (0.42; 2.45) | 0.54 (0.22; 1.30) | 0.97 (0.51; 1.85) | 0.74 (0.36; 1.53) | 0.61 (0.28; 1.33) | **2.07 (1.17; 3.68)*** |
| 4. Quartile | 0.32 (0.08; 1.22) | 0.41 (0.13; 1.28) | 0.74 (0.27; 1.96) | 0.82 (0.35; 1.94) | 0.73 (0.37; 1.43) | 1.03 (0.54; 1.97) | 0.95 (0.46; 1.96) | 1.24 (0.66; 2.34) |
| **10-year follow-up, age-adjusted** | | | | | | | | |
| continuous | **0.71 (0.56; 0.92)*** | 0.86 (0.49; 1.50) | 0.84 (0.55; 1.27) | **0.60 (0.38; 0.95)*** | 1.05 (0.81; 1.35) | 0.88 (0.61; 1.19) | 0.88 (0.68; 1.14) | 1.15 (0.91; 1.45) |
| 1. Quartile | Ref. | Ref. | Ref. | Ref. | Ref. | Ref. | Ref. | Ref. |
| 2. Quartile | 0.49 (0.13; 1.90) | 0.94 (0.23; 3.83) | 0.89 (0.22; 3.53) | 0.41 (0.12; 1.32) | 0.90 (0.40; 2.00) | 0.84 (0.41; 1.73) | 1.15 (0.55; 2.41) | 1.30 (0.65; 2.60) |
| 3. Quartile | 0.65 (0.17; 2.20) | 1.49 (0.40; 5.59) | 0.93 (0.23; 3.54) | 0.59 (0.19; 1.68) | 1.30 (0.65; 2.62) | 0.88 (0.46; 1.88) | 1.17 (0.56; 2.40) | 1.72 (0.88; 3.34) |
| 4. Quartile | 0.18 (0.02; 1.56) | 0.28 (0.02; 2.70) | 0.45 (0.08; 2.44) | 0.37 (0.10; 1.36) | 1.10 (0.51; 2.33) | 0.84 (0.40; 1.75) | 0.91 (0.40; 2.05) | 1.35 (0.64; 2.68) |
| **10-year follow-up, multivariable-adjusted** | | | | | | | | |
| continuous | 0.72 (0.51; 1.01) | 0.97 (0.59; 1.61) | 0.84 (0.46; 1.52) | 0.62 (0.36; 1.10) | 1.04 (0.80; 1.35) | 0.88 (0.60; 1.25) | 0.87 (0.66; 1.16) | 1.23 (0.95; 1.53) |
| 1. Quartile | Ref. | Ref. | Ref. | Ref. | Ref. | Ref. | Ref. | Ref. |
| 2. Quartile | 0.58 (0.14; 2.41) | 0.90 (0.21; 3.77) | 0.73 (0.16; 3.09) | 0.51 (0.15; 1.69) | 0.90 (0.40; 2.04) | 0.78 (0.37; 1.65) | 1.11 (0.51; 2.31) | 1.46 (0.75; 3.16) |
| 3. Quartile | 0.73 (0.15; 3.14) | 1.75 (0.45; 6.46) | 1.01 (0.24; 4.23) | 0.51 (0.16; 1.68) | 1.15 (0.56; 2.43) | 0.81 (0.45; 1.78) | 1.12 (0.54; 2.47) | 2.15 (1.05; 4.34) |
| 4. Quartile | 0.29 (0.03; 2.70) | 0.36 (0.04; 3.20) | 0.53 (0.09; 2.99) | 0.54 (0.13; 2.31) | 1.16 (0.54; 2.49) | 0.86 (0.41; 1.80) | 0.91 (0.39; 2.21) | 1.45 (0.69; 3.02) |

Data are relative risk and their 95% confidence interval with p < 0.05 marked as *****. Quartile 1 was used as reference.

The multivariable model was adjusted for age, body mass index, smoking status (three categories), alcohol consumption, physically inactivity, hypertension, and time of blood sampling. Longitudinal analyses were additionally adjusted for inverse probability weights for drop-out of baseline examination to follow-up and performed only in participants without depressive symptoms at baseline. SHBG, sex hormone-binding globulin; CI, confidence interval.
