# Supplementary material for: Associations of androgens with depressive symptoms and cognitive status in the general population
Source: PLoS One. 2017 May 12;12(5):e0177272. doi: 10.1371/journal.pone.0177272 (PMC5428943; doi:10.1371/journal.pone.0177272)
Supplement: S3 Table — (DOCX) [file pone.0177272.s003.docx]

**S3 Table**

**S3 Table :** Associations of sex hormones and SHBG with change in mini mental status in linear regression models, separately in men and women and additionally adjusted for time of blood sampling.

|  | **β-coefficients** **(95% CI)** | | | |
| --- | --- | --- | --- | --- |
|  | **Total Testosterone** | **Androstenedione** | **Free Testosterone** | **SHBG** |
| **Men** | | | | |
| **5-year follow-up** | | | | |
| age-adjusted | 0.11 (-0.10; 0.38) | 0.32 (-0.21; 0.89) | 0.04 (-0.15; 0.26) | **0.44 (0.11; 0.74)*** |
| multivariable-adjusted | 0.01 (-0.15; 0.20) | 0.36 (-0.12; 0.91) | -0.02 (-0.20; 0.19) | 0.24 (-0.11; 0.63) |
| **10-year follow-up** | | | | |
| age-adjusted | 0.01 (-0.21; 0.23) | 0.33 (-0.13; 0.80) | 0.07 (-0.16; 0.31) | 0.10 (-0.26; 0.47) |
| multivariable-adjusted | 0.01 (-0.22; 0.24) | 0.27 (-0.21; 0.75) | 0.05 (-0.20; 0.30) | 0.09 (-0.31; 0.49) |
| **Women** | | | | |
| **5-year follow-up** | | | | |
| age-adjusted | -0.03 (-0.43; 0.36) | -0.15 (-0.44; 0.16) | 0.23 (-0.18; 0.66) | -0.31 (-0.73; 0.10) |
| multivariable-adjusted | -0.11 (-0.48; 0.28) | -0.24 (-0.53; 0.10) | 0.23 (-0.17; 0.64) | -0.37 (-0.80; 0.04) |
| **10-year follow-up** | | | | |
| age-adjusted | 0.05 (-0.31; 0.50) | 0.01 (-0.31; 0.35) | 0.22 (-0.28; 0.83) | -0.10 (-0.48; 0.26) |
| multivariable-adjusted | -0.03 (-0.47; 0.41) | 0.01 (-0.37; 0.41) | 0.15 (-0.45; 0.78) | -0.05 (-0.44; 0.35) |

Data are β coefficients and their 95% confidence interval with p < 0.05 marked as *****.
